# Health Care Professionals’ Views on the Use of Passive Sensing, AI, and Machine Learning in Mental Health Care: Systematic Review With Meta-Synthesis

**DOI:** 10.2196/49577

**Published:** 2024-01-23

**Authors:** Jessica Rogan, Sandra Bucci, Joseph Firth

**Affiliations:** 1 Division of Psychology and Mental Health, School of Health Sciences, Faculty of Biology, Medicine and Health, Manchester Academic Health Sciences The University of Manchester Manchester United Kingdom; 2 Greater Manchester Mental Health NHS Foundation Trust Manchester United Kingdom

**Keywords:** artificial intelligence, machine learning, passive sensing, mental health care, clinicians, views, meta-synthesis, review, mental health, health care, health care professionals, psychology, psychiatry, mental health professionals, mobile phone

## Abstract

**Background:**

Mental health difficulties are highly prevalent worldwide. Passive sensing technologies and applied artificial intelligence (AI) methods can provide an innovative means of supporting the management of mental health problems and enhancing the quality of care. However, the views of stakeholders are important in understanding the potential barriers to and facilitators of their implementation.

**Objective:**

This study aims to review, critically appraise, and synthesize qualitative findings relating to the views of mental health care professionals on the use of passive sensing and AI in mental health care.

**Methods:**

A systematic search of qualitative studies was performed using 4 databases. A meta-synthesis approach was used, whereby studies were analyzed using an inductive thematic analysis approach within a critical realist epistemological framework.

**Results:**

Overall, 10 studies met the eligibility criteria. The 3 main themes were uses of passive sensing and AI in clinical practice, barriers to and facilitators of use in practice, and consequences for service users. A total of 5 subthemes were identified: barriers, facilitators, empowerment, risk to well-being, and data privacy and protection issues.

**Conclusions:**

Although clinicians are open-minded about the use of passive sensing and AI in mental health care, important factors to consider are service user well-being, clinician workloads, and therapeutic relationships. Service users and clinicians must be involved in the development of digital technologies and systems to ensure ease of use. The development of, and training in, clear policies and guidelines on the use of passive sensing and AI in mental health care, including risk management and data security procedures, will also be key to facilitating clinician engagement. The means for clinicians and service users to provide feedback on how the use of passive sensing and AI in practice is being received should also be considered.

**Trial Registration:**

PROSPERO International Prospective Register of Systematic Reviews CRD42022331698; https://www.crd.york.ac.uk/prospero/display_record.php?RecordID=331698

## Introduction

### Background

Mental health problems are highly prevalent globally, with approximately 1 in 8 people experiencing mental health difficulties, which can have significant personal and economic consequences [1]. Rapid growth in digital technology innovation has led to an increased interest in digital mental health interventions [2]. Digital tools with built-in sensors, such as smartphones, smartwatches, and other wearable devices, allow for the unobtrusive and continuous collection of objective data, providing insight into user behavior and physiology [3]. Machine learning, which is a branch of artificial intelligence (AI), can be applied to these data to *learn* from it and generate clinically actionable insights and predictions [4]. It has therefore been suggested that passive sensing data and applied machine learning methods could overcome what some describe as trial-and-error–driven approaches used in mental health care by supporting precise diagnoses and prognoses [5]. Indeed, mental health remains one of the only domains in health care that relies only on service users’ self-report of cognitive and emotional states and symptoms and on clinicians to accurately recognize and map these states to make diagnostic, prognostic, and therapeutic decisions [6]. Passive sensing data and AI may offer a means to overcome the pitfalls of current clinical measures by presenting a more complete picture of a person’s difficulties [7]. For example, raw sensor data captured regarding speech characteristics, location, and activity can be transformed to derive high-level behavioral markers, such as fatigue, sleep disruption, and mood, which can be used to identify clinical states, such as depression [8]. In addition, digital tools that allow for passive sensing can support service users’ self-management of symptoms and access to digitally delivered therapies [4]. Through self-management, service users may feel empowered [9], and service user and clinician access to digital remote data capture has the potential to identify early warning signs of deterioration, providing the opportunity to reduce the risk of relapse of mental health difficulties via early identification and intervention [10]. This may be particularly useful, as current health care systems generally rely on the delivery of treatment by scheduled appointments, which can result in warning signs of mental health relapse being missed or treated too late [11]. Using sensors from digital tools, such as smartphones and wearable devices, to identify clinical and behavioral features of worsening mental health and applying machine learning methods to identify patterns in the data could augment mental health care by delivering more precise treatment at the time it is needed [12].

Despite the potential benefits, there remains a persistent gap between the rapid developments in digitally supported mental health care and the successful adoption of these tools in clinical practice [13]. A key driver to the potential success of digitally supported health care uptake is the willingness, confidence, and capacity of clinicians to make changes to their practice [9]. Resistance to incorporating digital approaches in clinical practice can occur for various reasons, including the lack of technological literacy, fear that AI models could replace professionals, and concerns about ethical and legal issues [6]. There is trepidation that core aspects of clinician roles, such as diagnosis, assessment, formulation and treatment, may be delegated to AI models without human input [14]. This has been viewed as dehumanizing and could have negative implications for therapeutic relationship [15]. Ethical issues have also been raised, such as implications for service user privacy and data security [16]. As clinicians’ perceptions and attitudes pose a potential barrier to implementation [17], it is important that they are invited into the dialog around digitally supported AI in mental health care, to embrace any benefits there might be, as well as share their concerns and explore the limitations and risks [2]. However, it has been noted that stakeholder’s views are rarely considered in model design or evaluation in relation to machine learning approaches [18]. Indeed, professionals have felt that their knowledge and views have been disregarded in the design of digital health solutions or are only considered as an afterthought [19]. As the extent to which these methods can be successfully implemented in health care depends on their acceptability [3], research is needed to understand stakeholders’ perspectives on digital health systems [11].

### Objectives

Although there have been some qualitative studies exploring mental health care professionals’ views and experiences of passive sensing and AI in mental health care, there are no published reviews that systematically aggregate these findings, specifically through examining participants’ experiences and perspectives, both deeply (because of the qualitative approach) and broadly (because of the integration of studies from different health care contexts and participants) [20]. This meta-synthesis aims to synthesize and evaluate the relative strengths of the qualitative literature regarding mental health care professionals’ views on the use of passive sensing and AI in mental health care to provide a new, comprehensive interpretation of the findings that goes beyond the depth and breadth of the original studies [21]. Although research continues to grow in this area, it is now an appropriate time to review the literature, as the COVID-19 pandemic has increased the urgency for creating digital interventions that can fulfill the full potential of digital health [22], and it is necessary to engage multiple stakeholder groups early in the design and development process [23].

## Methods

### Overview

Meta-synthesis is a systematic review and integration of findings from qualitative studies to facilitate the transfer of knowledge and bring together a broad range of participants and descriptions [20]. A systematic approach for identifying and assessing the quality of potential papers, followed by analysis of the data and synthesis, was used with the aim of understanding what mental health care professionals think about the use of passive sensing and AI in mental health care. The review protocol was developed and registered with the International Prospective Register of Systematic Reviews (PROSPERO CRD42022331698).

### Eligibility Criteria

Eligible papers for this review (1) were peer-reviewed studies published in English that used a qualitative method—mixed methods studies were also included, but only the qualitative findings were considered; and (2) examined health care professionals’ views on hypothetical or actual use of service user-facing digital tools that use passive sensing and AI in mental health care. Studies with participants that included other stakeholders, as well as health care professionals, were not discounted; however, findings were only included if they were explicitly associated with mental health professionals. There were no limits on the publication year.

### Search Strategy

A discussion within the research team and a review of the literature allowed for the identification of common terminology used in this research area and the selection of search terms. The search tool “SPIDER” (sample, phenomenon of interest, design, evaluation, research type) was used to ensure that all relevant areas were covered when developing the search terms. Relevant studies were identified through systematic searches of the following electronic databases: AMED, PsycINFO, Embase, and Medline. The search terms were (clinician*) OR (health care professional*) OR (staff) OR (physician*) OR (provider*) OR (practitioner*) OR (psychologist*) OR (doctor*) OR (therapist*) OR (care coordinator*) OR (mental health nurse*) OR (psychiatric nurse*) OR (support worker*) OR (counsellor *) OR (case manager*) OR (GP*) AND (view*) OR (opinion*) OR (perception*) OR (qualitative) OR (interview*) AND (remote monitoring) OR (digital phenotyping) OR (machine learning) OR (passive sens*) OR (passive monitor*) OR (passive data) OR (artificial intelligence) OR (wearables). A manual search of references and citations from eligible articles was also performed by JR to identify additional studies. Papers were initially screened according to title and abstract, followed by a full article.

### Study Selection

**Figure 1:**
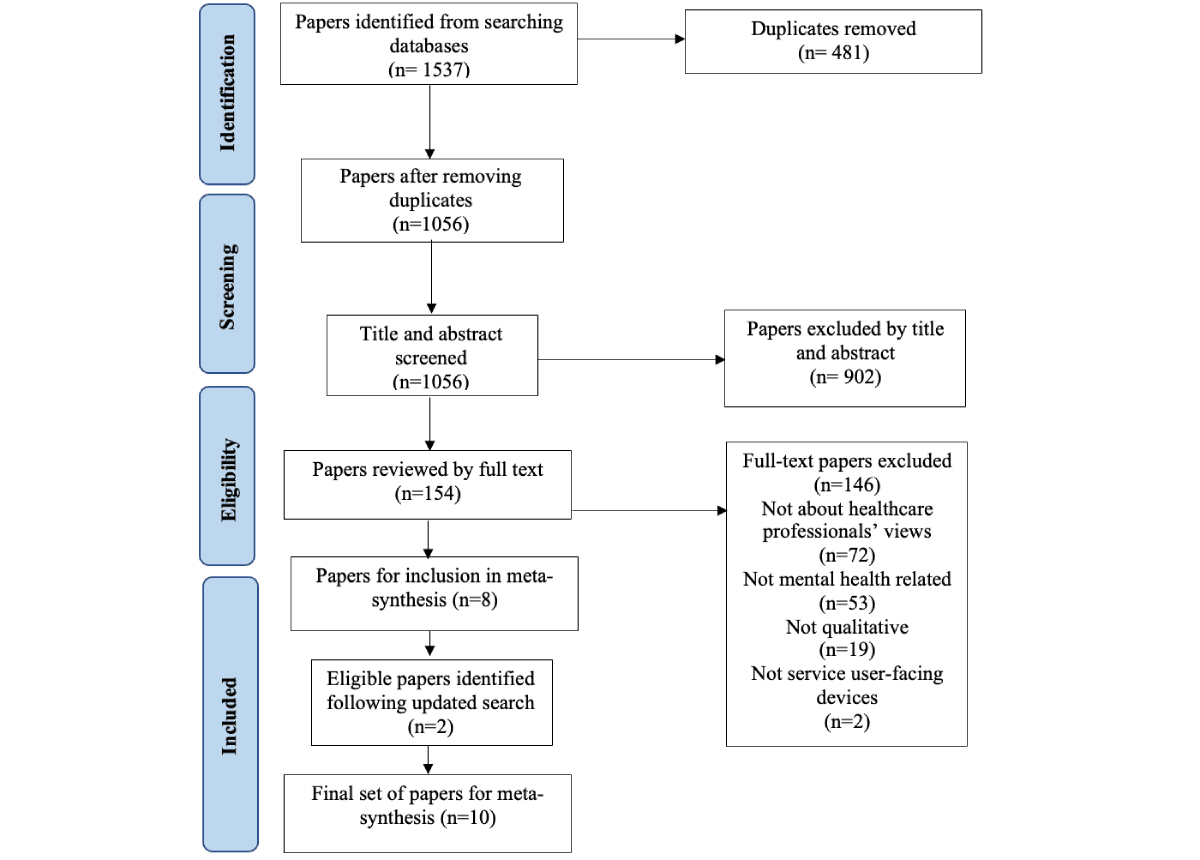
PRISMA (Preferred Reporting Items for Systematic Reviews and Meta-Analyses) flow diagram of systematic search.

The study selection and exclusion processes were conducted in accordance with the PRISMA (Preferred Reporting Items for Systematic Reviews and Meta-Analyses) guidelines [24] in October 2022 and are outlined in Figure 1. Article titles and abstracts were screened for eligibility by JR. If the inclusion criteria were unclear, full-text articles were obtained and reviewed. Any uncertainty regarding study eligibility was resolved through discussion with a wider research team. A second independent rater screened 10% (106/1056) of titles and reviewed 10% (16/154) of full-text articles to assess the reliability of the study selection. There was an “almost perfect” level of agreement between the raters at the screening stage (*k*=0.918) and at the full-text stage (*k*=1) [25]. As all studies were published in recent years, the search was conducted again in February 2023. Overall, 10 studies met the eligibility criteria and were included in the review.

### Quality Appraisal

Study quality was assessed using the Critical Appraisal Skills Programme (CASP) tool for quality appraisal in qualitative evidence synthesis (CASP, 2018, [26]), which assesses the strengths, limitations, relevance, and credibility of qualitative research. The CASP comprises 10 items that focus on different methodological aspects of qualitative studies, such as method, design, recruitment, data collection, and reflexivity. It is considered a good measure of transparency of research practice and reporting standards and is recommended for use in health-related research [27]; therefore, it was deemed appropriate for use in this review. A 3-point scale was used, with a score applied to each criterion (0=criterion not met, 1=criterion partially met, and 2=criterion totally met) [28]. Therefore, papers were given a total quality score of 20.

The scoring was completed by JR. A second independent rater assessed the quality of 50% (5/10) of the studies, and the scores were compared at the item level. Interrater reliability estimates showed good agreement between raters (*k*=0.832) [25]. Disagreements in ratings were resolved through discussion among raters until agreement was reached.

### Data Extraction and Synthesis

The included studies were read and reread to ensure that they met the inclusion criteria. Key study information was then recorded, including the number and characteristics of participants, aims of the research, analysis method used, and the settings (Table 1). In addition, the original authors’ analysis of primary qualitative data was extracted (second-order constructs), and individual participants’ quotes were also noted (first-order constructs), in line with meta-synthesis principles [29]. An inductive thematic analysis approach within a critical realist epistemological framework was then taken with the aim of developing a cohesive, synthesized understanding of the data [30] and new interpretations [31]. JR completed the coding of the text and quotes using NVivo qualitative data analysis software (NVivo version 12, Lumivero). The constructs were then grouped into core themes. These themes were discussed by a broader research team, considering how each paper contributed to each core theme. The themes were then grouped into final higher-order themes, which were again reviewed and agreed upon by the research team. These themes are considered third-order constructs and allow for reflection on how each paper’s findings fit within the wider literature and for findings to extend beyond the original papers [21]. JR returned to the papers to ensure that the themes identified reflected the data and that other themes were not overlooked.

**Table 1:** Summary of included studies.

Study	Participants	Aim	Data analysis	Setting	Results and themes	Critical Appraisal Skills Programme quality appraisal score (out of 20)
Greer et al [32] (the United Kingdom)	Five focus groups, made up of mental health nurses (N=25); age range 22-64 y; mean age 42.7 y; male: n=9; female: n=16	To explore staff views, specifically benefits and barriers to using remote monitoring to predict risk of inpatient aggression	Thematic analysis	Inpatient forensic mental health service, the United Kingdom	Utility in clinical practice, risk to user safety and well-being, data security and privacy, impact on staff workload, engagement, and adherence	17
Ng et al [33] (the United States)	Interviews with mental health professionals (N=17); age and gender not stated	Explore opportunities and barriers mental health staff perceive in applying sensor-captured patient-generated data among populations with PTSD^a^ in routine care settings and how providers perspectives inform the design of tracking systems and strategies to implement technologies	Thematic analysis	Centre for Veterans With PTSD, the United States	Patient-driven uses of Fitbit and its data; integrating Fitbit data into treatment protocols; challenges to the use in treatment	18
Blease et al [34] (global)	Web-based survey of psychiatrists (N=791); age range 25-≥65 y; mean age group 35-44 y; male: n=550; female: n=230; other: n=11	To explore psychiatrists’ opinions about the potential impact of innovations in AI^b^ and machine learning on psychiatric practice	Qualitative descriptive analysis of written responses	Web-based survey across 22 countries	Patient-psychiatrist interactions, quality of patient medical care, profession of psychiatry, health systems	18
Thenral and Annamalai [35] (India)	Interviews with psychiatrists (n=14), patients (n=14), technology experts (n=13), and chief executive officers (n=5); overall (N=46); psychiatrist characteristics: mean age 35.5 y; male: n=7; female: n=7	To understand the perceived challenges in building, deploying, and using AI-enabled telepsychiatry for clinical practice	Grounded theory	Practices in urban areas of India: Chennai, Mumbai, Bangalore, and Delhi	Knowledge and gaps deficit; attitudes and perception; data challenges; ethical, legal accountability; AI related; health system infrastructure; human resources and skills; technology; clinical practice	15
Dawoodbhoy et al [36] (the United Kingdom)	Interviews with health care professionals (n=9) and AI experts (n=11); overall (N=20); age and gender of health care professionals not stated	To identify issues in patient flow on mental health units and align them with potential AI solutions, ultimately devising a model for their integration at service level	Thematic analysis	Acute mental health inpatient units, the United Kingdom	Current mental health inpatient service and patient flow model: patient factors; problems with social care; problems with clinical management; problems with inpatient service and system; solutions	16
Rodriguez-Villa et al [23] (the United States and India)	Focus groups and interviews with mental health clinicians (n=53) and service users and their families *(*n=75); overall (N=128); clinician characteristics: age range 23-72 y; mean age 36 y; gender not stated	To engage clinicians and people living with schizophrenia spectrum disorders and their family members from 3 study sites distinct in culture and setting in developing new features and co-designing the mindLAMP app	Thematic analysis	Mental health services in the United States and India	COVID-19 led to an uptake of virtual therapy, presenting new challenges and opportunities for providers and people with schizophrenia, using technology; access to data may offer providers and people with schizophrenia new insight into illness and treatment, but too much data elicit discomfort; relevance and integrated experience increase engagement	18
Byrne et al [10] (Australia)	Focus groups with service users (n=12) and interviews with mental health clinicians (n=10); overall (N=22); clinician characteristics: age range 25-60 y; male: n=2; female: n=8	Explore patient and clinician-related acceptability of an mHealth^c^ device to monitor stress for severe mental illness	Thematic analysis	Community youth mental health service, Australia	Self-monitoring improves insight; clinician monitoring as a benefit to treatment; privacy and data misuse concerns; ease of use; engaging design; procedural guidelines	20
de Angel et al [3] (the United Kingdom)	Three focus groups: 2 focus groups with patients (n=16) and 1 focus group with mental health clinicians (n=6); overall (N=22); characteristics of clinicians: mean age 36.7 y; male: n=1; female: n=5	To identify clinically meaningful targets for digital health research and to explore patient and clinician attitudes toward the use of remote monitoring technologies and identify any perceived barriers to and facilitators of using these methods in psychological treatments for depression	Thematic analysis	Improving Access to Psychological Therapies services, the United Kingdom	Promoters of change (internal and external); markers of change (internal and external)	14
Gotzl et al [37] (Germany)	Two focus groups with young people (n=8) and interviews with experts, including psychologists (n=2); overall (N=13); age and gender of psychologists not stated	To investigate the subjective needs, attitudes, and preferences of key stakeholders toward an AI-informed mHealth app	Mixed methods-only qualitative component considered: content analysis	This study formed part of the living laboratory “AI4U-Artifical Intelligence for personalized digital mental health promotion and prevention in youth”	Young peoples’ understanding of mental health; experts understanding of mental health in youth; opportunities and risks seen by experts; experts’ recommendations	17
Reis and Maier [38] (Germany)	Interviews with mental health professional (N=15); mean age 35 y; male: n=9; female: n=6	To explore the application scenarios for artificial intelligence in mental health from the mental health professionals’ perspective and to evaluate the implementation readiness of scenarios	Content analysis	Mental health facilities in Germany	Application scenarios of AI in mental health: preselection and scheduling; patient monitoring during waiting times; documentation of treatment sessions; diagnosis support; relapse prophylactics; emergency care	16

^a^PTSD: posttraumatic stress disorder.

^b^AI: artificial intelligence.

^c^mHealth: mobile health.

## Results

### Summary of Papers

A total of 10 papers were deemed eligible for inclusion in this review. A total of 3 studies were conducted in the United Kingdom, 1 in India, 1 in the United States, 1 across both the United States and India, 1 in Australia, 2 in Germany, and 1 in a global study. In total, 6 studies used thematic analysis, 1 used a grounded theory approach, 1 used qualitative descriptive analysis, and 2 used content analysis. Participants in 4 of the papers were health care professionals only, with the remaining 6 papers including health care professionals as well as other stakeholders, such as service users and their families, technology experts, and technology company owners. The findings were only included if they were explicitly associated with health care professionals. The number of health care professionals ranged from 2 to 53 (mean 17). The age of the mental health professionals where this was reported (6 papers) ranged from 22 to 72 years. Among the 5 papers that reported gender, 28 participants were male, and 42 were female. Owing to the high number of participants in the global web-based survey [34], these data are described separately, with 791 participants taking part, ranging in age from 25 to ≥65 years. Of the participants, 550 identified as male, 230 identified as female, and 11 identified as others.

### Study Quality

The overall CASP quality appraisal scores are included in Table 1, and a breakdown of these scores is provided in Table 2. There was variation in the scores across the papers. Those given stronger scores tended to provide more detail as to why certain qualitative approaches were selected over others, provided details regarding the sample including why participants may have opted not to take part, and ethical considerations were reported. It should be noted that although some studies did make some reference to the relationship between researcher and participants, this was the area that scored lowest, with few studies referencing bias and considering the influence their own roles may have had on results and reporting. For papers that included other stakeholders alongside mental health professionals, higher scores were given if the results were written to clearly distinguish which themes were associated with which participant group.

**Table 2:** Quality ratings on each of the Critical Appraisal Skills Programme domains.

Study	Aims	Method	Design	Recruitment	Data collection	Bias and reflexivity	Ethical issues	Data analysis	Clear findings	Value
Greer et al [32]	2	2	1	2	2	0	2	2	2	2
Ng et al [33]	2	2	2	2	2	1	1	2	2	2
Blease et al [34]	2	2	1	2	2	1	2	2	2	2
Thenral and Annamalai [35]	2	2	2	2	2	0	0	2	1	2
Dawoodbhoy et al [36]	2	2	2	2	1	1	1	2	1	2
Rodriguez-Villa et al [23]	2	2	2	2	2	0	2	2	2	2
Byrne et al [10]	2	2	2	2	2	2	2	2	2	2
de Angel et al [3]	2	2	2	1	1	0	1	2	1	2
Gotzl et al [37]	2	2	2	1	2	1	2	2	1	2
Reis and Maier [38]	2	2	2	2	2	0	0	2	2	2

### Findings

**Figure 2:**
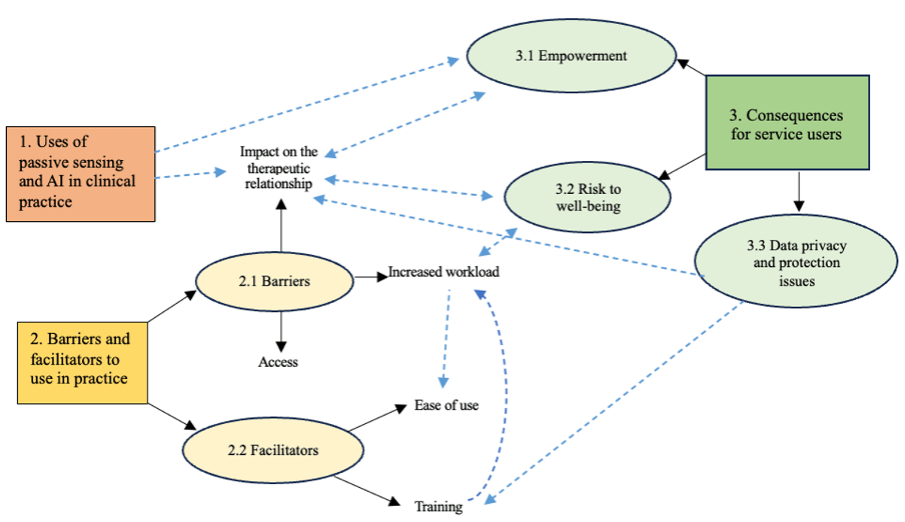
Themes, subthemes, and the relationships between them. AI: artificial intelligence.

Analysis of the data revealed three distinct but interrelated themes: (1) the use of passive sensing and AI in clinical practice, (2) barriers to and facilitators of use in practice, and (3) consequences for service users. A total of 5 subthemes were identified from the data. The themes, subthemes, and relationships between them are summarized in Figure 2.

### Theme 1: Uses of Passive Sensing and AI in Clinical Practice

Findings across the reviewed papers included how clinicians felt they could use passive sensing and AI in their practice. Passive sensing technology has been shown to be particularly useful, as the unobtrusive collection of objective service user data could offer a new information source [23]. This could provide insight into factors such as speech, social media use, and activity levels, which could be considered alongside self-report questionnaires and assessment tools to facilitate more accurate assessment of service users’ mental health difficulties [35]. In assessing service user needs, it was also felt that these data could clarify discrepancies between self-report, observation, and psychometrics and validate service users’ concerns [33]. It has been suggested that passive sensing data and AI could entirely replace some methods of assessment, such as questionnaires, to improve the clinical experience for both service users and clinicians [3,38]. Passive sensing data can reduce errors and biases in clinical decisions regarding diagnosis, treatment, and medication [34], as data-driven technologies may uncover correlations that humans cannot [36]:

The benefits would be greater reliability in diagnosis and prognosis, being able to choose specific customized treatment plans after analysis.

Participant in Blease et al [34]

Further suggestions were made as to how passive sensing and AI could be useful in therapeutic work, such as guiding productive discussions [33], setting treatment goals, delivering low-intensity support [3], tracking the efficacy of brief interventions [23], and encouraging ongoing engagement and regular self-reflection [38]. Furthermore, discussions were conducted about how AI’s ability to process, connect, and make conclusions from large amounts of data could be used to risk-stratify service users according to their personal factors and needs [36] and support identification and awareness of early warning signs, thus reducing the risk of relapse of mental health difficulties [32-34,37]. As clinicians have access to these data, it was also felt that they could identify when to intervene [38], which may further reduce a service user’s risk of deterioration in mental health [10]. Indeed, clinicians have reported that seeing a change in the data regarding a service user’s speech and self-care habits would promote awareness of a decline in their well-being [3]. This was considered useful in community and ward environments, where staff may not always have eyes on service users [32], particularly for those who may lack insight into their difficulties or do not volunteer information themselves [23,32]:

...not all our patients will be able to say, “oh well I feel agitated” or be able to come out and say it, but within themselves all the physical, you know, changes are taking place so I think it’s good, it will help us to see the covert, you know, things that are not outward that the patients cannot express.

Participant in Greer et al [32]

The idea that aspects of psychiatric work could gradually be replaced by AI was viewed as positive progression by some, but others were concerned that overreliance on AI and technology in practice may result in staff becoming deskilled [34]:

May lead to less skilled mental health staff.

Participant in Blease et al [34]

### Theme 2: Barriers and Facilitators to Use in Practice

#### Overview

Throughout the papers, participants discussed the perceived barriers to and facilitators of using passive sensing and AI in mental health care. The barriers discussed included access, concerns about clinicians’ workloads, and the potential negative impact on the therapeutic relationship. Facilitators included ease of use and training.

#### Subtheme 2.1: Barriers

##### Access

It was highlighted that technology is now readily available, and this was reported as a benefit to using passive sensing and AI in mental health practice as it may improve service user access to mental health care [34]. However, not all mental health services have sufficient access to technology [37] because of factors such as cost and the lack of the necessary infrastructure to support digital tools [3]. For example, participants reported that in India, most hospitals do not have access to the internet [35], and service users do not always have access to smartphones [23]. This would likely present significant barriers to health care professionals using such technology in mental health care. Therefore, it is important to consider the digital divide, that is, the gap between those who benefit from the digital age and those who do not [3]:

All these devices, technology, AI, etc., require high-speed internet...Majority of the hospitals do not have internet...patients who have basic livelihood issues cannot afford a device or internet.

Participant in Thenral and Annamalai [35]

##### Increased Workload

A further barrier discussed was the impact of passive sensing and AI could have on clinicians’ workloads. Clinicians wondered about the amount of time and effort required to incorporate data flows into their practice and whether they would be required to review data before sessions [33], which could result in clinicians trawling through a significant amount of data to generate actionable insights [3]. Indeed, participants reported feeling “overwhelmed” when presented with passively collected data [23]:

I feel overwhelmed with the data to begin with.

Participant in Rodriguez-Villa et al [23]

Queries were also raised around documentation and whether the use of these tools would increase administrative work for clinicians [33,34]. Furthermore, it was suggested that clinicians may have to spend time with service users reviewing the use of devices and verifying data, which could take time away from evidence-based practices. If clinicians are alerted to changes in behavior that are the result of inaccurate readings, this could cause unnecessary alarm and waste clinicians’ time [33]:

There’s always so much to do...You’re already kind of preparing for sessions, and at a certain point it’s like, “How much am I treating or assessing what I’m seeing on a screen or on paper compared to just talking to someone and figuring stuff out together?”

Participant in Ng et al [33]

Managing risk was another concern raised, with participants wondering about their clinical responsibility for monitoring the data for risk issues [3,10,35] because responding to constant data streams would not be possible [23]. This is important because risk aversion is cited as a potential barrier to engagement [36].

In contrast, it has been suggested that passive sensing and AI have the potential to multiply resources, in that it offers a means of support to service users when health care professionals are unavailable [38]:

Having a system that treats people would be awesome. We cannot be everywhere, and the number of mental health professionals is too low.

Participant in Reis and Maier [38]

##### Impact on the Therapeutic Relationship

An issue that arose across studies was the impact that use of passive sensing and AI in practice could have on the therapeutic relationships. Some service users may prefer in-person consultations [35]; therefore, using digital tools and AI methods as a replacement of human contact could be determinantal to the therapeutic alliance [3] because of a loss of empathy and inaccurate interpretations of service users’ presentations [34]:

Psychiatry is incompetent and incomplete without empathy. I doubt a machine could ever empathise with a live human being...I don’t think affect of patient and mood, feelings, emotions can be analysed accurately.

Participant in Blease et al [34]

A potential lack of meaningful interactions between service users and clinicians [33] has led some to believe that service users may become resistant to or refuse treatment [34]. It was also suggested that, as service users are less accountable to clinicians, this could negatively impact motivation [3]. Furthermore, service users may become reliant on a device during treatment, and having this subsequently removed could have negative repercussions, including service users becoming mistrusting of services [3]. It was also highlighted that clinicians may not be able to fully trust the data they receive, as participants suggested that service users may influence these data deliberately [34]:

If a patient simulates a disease AI might not be able to determine it.

Participant in Blease et al [34]

Having said this, it was proposed that allowing service users to submit data to clinicians, who could then respond with recommendations, would enable remote support and continuity of care, which could strengthen the therapeutic relationship [23]. It appeared that the general consensus was that although digital tools may enhance practice, they should not replace service user or staff interactions, something which was viewed as integral to the therapeutic relationship [32].

#### Subtheme 2.2: Facilitators

##### Ease of Use

Throughout the studies, it was highlighted that to improve engagement with digital tools that allow for passive sensing and applied AI methods, the technology and systems would have to be relatively straightforward to use in terms of accessibility and convenience [3]. Health care professionals discussed that clinicians are very busy and would not have time to navigate complicated systems [10]:

I think it needs to be relatively simple...not over complicated and very easy to navigate.

Participant in Byrne et al [10]

To ensure ease of use, suggestions were made, such as including relevant stakeholders in the development of such technologies and related systems to ensure that they use accessible language [37] and presenting the data in a simple way that is easy to understand [3]:

Being able to have a really simple, easy way to compare the progress throughout the weeks of treatment. So, you would obviously be collecting a huge amount of data but if there was a way that we could somehow get, “Okay, you did an average of X amount of steps in week one, and your average sleep was X amount with waking up X amount of times.”

Participant in de Angel et al [3]

##### Training

The importance of training to support clinicians in using digital tools, passively collected data, and applied AI methods in practice has been emphasized in most studies. This was discussed in the context of being given time to access the training as well as time to consider implementation [3]:

As long as we’ve had adequate training...And it’s not just having the training, it’s then having the time to think about that afterwards and incorporate it into your practice which would require a corresponding decrease in clinical work.

Participant in de Angel et al [3]

Clinicians may differ in technological literacy, and some may generally find technology challenging [35]. It was discussed that this technology will only be useful if clinicians understand it and feel comfortable using it [23]. For training to be adequate, it was suggested that clinicians would value clear procedures and guidance on when and how these digital technologies should be used [10], how to connect the data to their established clinical practice [33], and how to interpret data. Depending on the condition, certain markers of behavior could be interpreted positively or negatively [3]. Clinicians would also require clear guidelines regarding responsibility, interoperability, information governance, and potential risks [36]:

You’re going to need [to] train them on how to balance all of these different pieces of data they have access to and how to prioritize the data. I think it would be especially important for new therapists coming on. It would probably be pretty overwhelming for some to have access to that much data and I think we would need to do like a standard operating procedure of how to [deal with] the information.

Participant in Ng et al [33]

Clinicians would further benefit from being informed of the evidence base around passive sensing and AI in mental health care [33], especially as the belief that there is a lack of studies to support the use of AI technology in health care could be a barrier to clinician engagement [35]:

There is also a lack of well-established trials and studies to understand the applicability of AI-related technology. They build solutions with no real-time applications.

Participant in Thenral and Annamalai [35]

### Theme 3: Consequences for Service Users

#### Overview

Throughout the studies, findings on the consequences that passive sensing and AI in mental health care could have for service users were discussed. There appeared to be a positive notion that this could empower service users, although it was also acknowledged that there could be risks to service users’ well-being. Concerns have also been raised regarding the protection and safety of service users’ data.

#### Subtheme 3.1: Empowerment

It was suggested that passive sensing and AI could facilitate “knowledge transfer” and empower service users to understand how their actions, feelings, and thoughts are intertwined [37]. By increasing insight into mental health, self-monitoring allows service users to respond to symptoms and take action themselves [10]. Service users’ monitoring and managing their mental health may involve connecting with other users and supporting one another, setting reminders to take medication, and responding to prompts to engage in helpful strategies [3]. Thus, service users’ ability to monitor their mental health–related data can be empowering [33]:

A lot of the thoughts you have are that you are incapable, inadequate, cannot accomplish things. So, that [data] kind of directly speaks against that, right? “I am able to accomplish something, like reaching 10,000 steps a day.

Participant in Ng et al [33]

This self-management could increase awareness of early warning signs, reduce the risk of relapse, and therefore decrease demand for services, for example, by reducing hospital admissions [36].

#### Subtheme 3.2: Risk to Well-Being

In contrast, concerns have been raised about the accuracy of sensing technology, as making health care decisions based on unreliable sensors could potentially be harmful [3]. In addition, if service users have access to their health data, this could cause some to become hyperfocused on their data, catastrophize, or become disheartened by lack of progress or negative trends [33]. This was thought to be particularly pertinent to those who experience health anxiety [3] or paranoia, as “tracking” behavior could exacerbate symptoms [32]:

When you give this to a paranoid patient, they will think you are monitoring them. It will be so difficult to explain it to them to understand it that this is what you’re monitoring...This paranoia could also lead to them not even wearing this.

Participant in Greer et al [32]

Ng et al [33] highlighted the importance of clinicians, suggesting alternate ways for service users to frame or interact with their data. This may be important in ensuring that recommendations delivered to service users do not arouse false expectations of users [37]:

I would see a risk if the app claimed: “if you go through these ten steps...then you are a different person” [laughs]...A good app would be characterized by the fact that the user does not internalize a problem centred perspective, but that he...gets the feeling: “I am okay.”

Participant in Gotzl et al [37]

Another concern highlighted by participants working in mental health wards was that service users could harm themselves using a digital device. For example, if an armband that stretches it could be used as a ligature, the design of devices is an important consideration [32]:

...how far does it stretch, can you put it round your neck? Well, that might be an issue, you know, ligatures.

Participant in Greer et al [32]

...should be something that they cannot use as a weapon, like, there shouldn’t be any metal or something that they can use to self-harm. [Participant in Greer et al]

Participant in Greer et al [32]

#### Subtheme 3.3: Data Privacy and Protection Issues

Participants reported that in practice, they will often recommend apps to service users without reviewing privacy policies, citing a lack of time as the reason for not investigating this further [23]. However, in most studies, concerns have been raised regarding privacy in relation to passive sensing data. It has been suggested that the collection of personal data through digital devices that allow passive sensing could increase the risk of loss of confidentiality and misuse of data [34,36], which could negatively impact therapeutic relationships [10]. In line with this, it was felt that service users would have less control over what they chose to share, which may feel uncomfortable for service users and lead clinicians to feel as though they are invading their privacy [10,23]:

...it does feel like it is personal information and to share all the details about their sleep and their activity levels—that could be quite tricky for some of them to share openly and knowing that we kind of can access it without them knowing or without them being there.

Participant in Byrne et al [10]

Data management was therefore seen as an important consideration, and it was highlighted that service users should be given choice over what they share and be made aware of who could access the data, what will happen if their data are leaked [3], how their data will be kept private and secure, and what their data will be used for [10]:

I think it’s about having a conversation with the client at the beginning about boundaries really, and once it’s clearer, how the information will be shared and can be shared, then you can...Normally put in those boundaries and then you can understand those concerns.

Participant in de Angel et al [3]

It is important to ensure that service users have capacity when making these decisions, as mental states can change and influence decision-making [32].

um, making sure they understand completely, cos some people are more paranoid on days...than other days so it could be they’re fine for 5 days then the sixth day they’re really paranoid.

Participant in Greer et al [32]

## Discussion

### Principal Findings

Across the papers reviewed, multiple ways in which passive sensing technology and applied AI methods could augment mental health care were identified, such as supporting service users in managing their mental health, improving diagnostic accuracy, monitoring treatment trajectories, and increasing access to timely support, thereby reducing the risk of relapse of mental health difficulties. Indeed, research has shown that passive data and AI methods have the potential to provide insight into service user behavior outside the clinic environment and provide real-time detection of behavioral anomalies, which could allow early identification and intervention before a deterioration in mental health [39]. However, despite the potential benefits, concerns have been raised that clinicians could become overreliant on digital technology in practice [40]. This could have negative consequences, as participants discussed that they may not be able to fully trust the data they receive because of service user influence and inaccurate sensors. Therefore, overreliance on inaccurate data can lead to misdiagnoses or missed diagnoses. Thus, decision-making should not be delegated to technology alone [41], and it is important for clinicians to acknowledge the limitations of objective data collection and applied AI methods to avoid tension between service users and clinicians [42]. This is particularly important, as research has shown that discrepancies between experience and tracking data can lead to upset, confusion, and disengagement [43], which may negatively impact the therapeutic relationship.

The influence that the use of passive sensing and applied AI methods could have on the therapeutic relationship was further discussed across papers. Although service users should feel empowered to make choices and manage their own mental health, access to human in-person support is deemed necessary. This reflects concerns that the use of AI in health care could lead to neglect of the therapeutic aspects of in-person consultation, such as consideration of motivation and self-advocacy, attendance to nonverbal cues, and social connection that can be provided by in-person clinical contact [44]. Fears were further raised that the absence of a therapeutic relationship may lead service users to disengage or refuse mental health care altogether. Research suggests that a therapeutic alliance can exist between a person seeking change and a change agent, which does not necessarily have to be a human health care professional, with digital tools and apps themselves having the potential to act as change agents [45].

Concerns have been raised across studies that service users may notice a decline in their mental health if they were to monitor aspects of their behavior and interpret subsequent passively collected data in such a way that increases anxiety or results in demotivation. Research has shown that tracking behavior can reduce enjoyment in walking-based activities [46] and increase eating disorder symptomology [47]. However, research has also found that the use of digital devices that allow passive sensing, such as wearables, can be a positive experience, with multiple psychological benefits identified by users, including increased sense of motivation and accountability [48]. Individual differences are therefore important for clinicians to consider, as certain characteristics may impact a service user’s ability to interpret their health data in a helpful way. For example, research has shown that high health literacy supports the understanding of passively collected health data and how to use it to work toward goals [49].

Across studies, clinicians discussed the impact that use of passive sensing and AI could have on their workload. Concerns appeared to be around reviewing significant amounts of data to identify clinically relevant information and risk monitoring. However, previous research has suggested that AI may in fact reduce clinicians’ workloads, as less time will be required to read through notes to understand a service user’s history, particularly because certain AI methods, such as natural language processing, could be applied to patient notes to summarize important information [50]. Furthermore, machine learning methods can facilitate work by highlighting previously inaccessible or less understood symptoms and patterns [6]. It has also been suggested that data received by clinicians regarding a service user’s behavior may allow them to identify those most in need of support and prioritize their workload, thus using their time more effectively [51]. To reduce concerns about increased workload, it would be useful for clinicians to receive data in a user-friendly format, allowing seamless access to relevant information. If devices and associated systems are not considered user-friendly and there are multiple technical issues, this will likely result in frustration and reluctance to engage [52]. Along with ease of use, training was discussed as a means to encourage clinicians to engage with devices that allow passive sensing and applied AI methods in their practice. Ways to make training useful for clinicians included ensuring that clinicians have access to clear guidance around incorporating data flows into their practice, managing risk issues, and data privacy and protection procedures. The latter is especially pertinent, as concerns about data security were a reoccurring theme throughout studies. Transparent guidelines will need to be developed, and codes of practice enforced around storage, ownership, and sharing of data [52]. However, it has been suggested that concerns about confidentiality of data may always remain; therefore, to facilitate engagement, the perceived value to clinicians and service users will need to outweigh these concerns [53]. As discussed in the reviewed studies, training should involve increasing awareness of the evidence base so that clinicians can understand the cost-benefits of engaging in passive sensing and AI in practice.

The final key issue is access. As highlighted in this review, access to technology could pose a barrier to engagement at both the service user and clinician level. For example, studies conducted in India have highlighted that not all hospitals offering mental health care have access to the internet. Therefore, considering the digital context within low- and middle-income countries, it is important to create digital-based mental health interventions intended for a global rollout. Indeed, Lee et al [54] highlighted that methods such as machine learning have the potential to advance health equity by supporting opportunities for equality in patient outcomes, performance, and resource allocation.

### Strengths and Limitations

Meta-synthesis allows for greater scope and generalizability than individual primary studies [55]. However, as data are transposed into third-order constructs, there is potential for the findings to move away from the empirical, conceptual, and theoretical contexts of primary qualitative studies [56]. Of the papers included, 2 used content analysis, which is a more descriptive approach to coding and data interpretation (Vaismoradi et al [57]). Thus, the findings may have been more heavily influenced by studies that used more robust qualitative methods, such as thematic analysis, which can provide a more detailed and nuanced account of the data (Braun and Clarke [58]). Furthermor e, the process and methods of meta-synthesis are heavily influenced by the focus and expertise of the authors, meaning that some concepts and theories may not have been considered. This limitation was managed through discussion with the research team on coding and themes as well as remaining attuned to personal perspectives that could introduce bias [59].

Efforts were made to include all eligible studies in this review and to avoid neglecting potentially important findings, such as checking the reference lists of all papers and searching the databases again at a later date to identify further studies that might have been published. However, it is possible that some studies were overlooked, particularly as the terminology in this research area can be diverse and studies were only included if they were published in the English language in peer-reviewed journals, meaning that important contributions to the literature may have been missed because of language and publication bias. The included studies were conducted across different mental health settings, such as primary care and inpatient settings, and across different countries. It is important to note that the health care systems and services within them differ globally, so the generalizability of the results may be limited. However, meta-synthesis of qualitative studies can transform findings into highly abstracted and generalizable theoretical frameworks [21].

### Future Directions

Considering the findings from this review and wider research in this area, a key barrier to implementing digital technology innovations is end user perceptions rather than technology innovation itself [3]. Therefore, it will be important for future research to gain a deeper understanding of service user views as well as other stakeholders, such as policy makers. Further research into the efficacy of passive sensing and AI in mental health care is necessary to build an evidence base that would support the scaling up of these approaches to routine service delivery. Real-world studies implementing passive sensing and AI in practice are needed to understand the contextual factors that impact uptake, which will be useful to gain knowledge that can support the development of implementation frameworks [60].

### Clinical Implications

These findings suggest that although clinicians are open-minded about the use of passive sensing and applied AI methods in mental health care, factors such as service user well-being, clinicians’ workloads, and the therapeutic relationship need to be considered. It is important to involve both service users and clinicians in the development of digital technologies and systems to ensure their ease of use. The development of policies, training, and clear guidelines on the use of passive sensing and AI in mental health care, including risk management and data security procedures, will also be key to facilitating clinician engagement and wide-scale adoption. Means for clinicians and service users to provide feedback on how the use of passive sensing and AI in practice is being received should also be considered, allowing reflection on any impact there might be on the therapeutic relationship, service user well-being, and clinicians’ workloads.

## Abbreviations

AI: artificial intelligence
CASP: Critical Appraisal Skills Programme
PRISMA: Preferred Reporting Items for Systematic Reviews and Meta-Analyses

## References

[1] (2022). World mental health report: transforming mental health for all. World Health Organisation.

[2] Wilson RL, Higgins O, Atem J, Donaldson AE, Gildberg FA, Hooper M, Hopwood M, Rosado S, Solomon B, Ward K, Welsh B (2023). Artificial intelligence: an eye cast towards the mental health nursing horizon. Int J Ment Health Nurs.

[3] de Angel V, Adeleye F, Zhang Y, Cummins N, Munir S, Lewis S, Laporta Puyal E, Matcham F, Sun S, Folarin AA, Ranjan Y, Conde P, Rashid Z, Dobson R, Hotopf M (2023). The feasibility of implementing remote measurement technologies in psychological treatment for depression: mixed methods study on engagement. JMIR Ment Health.

[4] Torous J, Bucci S, Bell IH, Kessing LV, Faurholt-Jepsen M, Whelan P, Carvalho AF, Keshavan M, Linardon J, Firth J (2021). The growing field of digital psychiatry: current evidence and the future of apps, social media, chatbots, and virtual reality. World Psychiatry.

[5] Chekroud AM, Bondar J, Delgadillo J, Doherty G, Wasil A, Fokkema M, Cohen Z, Belgrave D, DeRubeis R, Iniesta R, Dwyer D, Choi K (2021). The promise of machine learning in predicting treatment outcomes in psychiatry. World Psychiatry.

[6] Koutsouleris N, Hauser TU, Skvortsova V, De Choudhury M (2022). From promise to practice: towards the realisation of AI-informed mental health care. Lancet Digit Health.

[7] Fisher CE, Appelbaum PS (2017). Beyond googling: the ethics of using patients' electronic footprints in psychiatric practice. Harv Rev Psychiatry.

[8] Mohr DC, Zhang M, Schueller SM (2017). Personal sensing: understanding mental health using ubiquitous sensors and machine learning. Annu Rev Clin Psychol.

[9] Spadaro B, Martin-Key NA, Bahn S (2021). Building the digital mental health ecosystem: opportunities and challenges for mobile health innovators. J Med Internet Res.

[10] Byrne S, Tohamy A, Kotze B, Ramos F, Starling J, Karageorge A, Bhattacharyya T, Modesto O, Harris A (2022). Using a mobile health device to monitor physiological stress for serious mental illness: a qualitative analysis of patient and clinician-related acceptability. Psychiatr Rehabil J.

[11] Bucci S, Lewis S, Ainsworth J, Haddock G, Machin M, Berry K, Berry N, Edge D, Emsley R (2018). Digital interventions in severe mental health problems: lessons from the Actissist development and trial. World Psychiatry.

[12] Zhang X, Lewis S, Chen X, Berry N, Bucci S (2022). Mental health professionals views and the impact of COVID-19 pandemic on implementing digital mental health in China: a nationwide survey study. Internet Interv.

[13] Fiske A, Henningsen P, Buyx A (2019). Your robot therapist will see you now: ethical implications of embodied artificial intelligence in psychiatry, psychology, and psychotherapy. J Med Internet Res.

[14] Rodriguez F, Scheinker D, Harrington RA (2018). Promise and perils of big data and artificial intelligence in clinical medicine and biomedical research. Circ Res.

[15] Chancellor S, De Choudhury M (2020). Methods in predictive techniques for mental health status on social media: a critical review. NPJ Digit Med.

[16] Rubeis G (2022). iHealth: the ethics of artificial intelligence and big data in mental healthcare. Internet Interv.

[17] Berry N, Lobban F, Bucci S (2019). A qualitative exploration of service user views about using digital health interventions for self-management in severe mental health problems. BMC Psychiatry.

[18] Wiens J, Saria S, Sendak M, Ghassemi M, Liu VX, Doshi-Velez F, Jung K, Heller K, Kale D, Saeed M, Ossorio PN, Thadaney-Israni S, Goldenberg A (2019). Do no harm: a roadmap for responsible machine learning for health care. Nat Med.

[19] Wilson R (2020). Why nurses need to be at the centre of new developments in digital technology. Nurse Res.

[20] Lachal J, Revah-Levy A, Orri M, Moro MR (2017). Metasynthesis: an original method to synthesize qualitative literature in psychiatry. Front Psychiatry.

[21] Mohammed MA, Moles RJ, Chen TF (2016). Meta-synthesis of qualitative research: the challenges and opportunities. Int J Clin Pharm.

[22] Hasson-Ohayon I, Lysaker PH (2020). Special challenges in psychotherapy continuation and adaption for persons with schizophrenia in the age of coronavirus (COVID-19). Counsel Psychol Q.

[23] Rodriguez-Villa E, Rozatkar AR, Kumar M, Patel V, Bondre A, Naik SS, Dutt S, Mehta UM, Nagendra S, Tugnawat D, Shrivastava R, Raghuram H, Khan A, Naslund JA, Gupta S, Bhan A, Thirthall J, Chand PK, Lakhtakia T, Keshavan M, Torous J (2021). Cross cultural and global uses of a digital mental health app: results of focus groups with clinicians, patients and family members in India and the United States. Glob Ment Health (Camb).

[24] Moher D, Liberati A, Tetzlaff J, Altman DG, PRISMA Group (2009). Preferred reporting items for systematic reviews and meta-analyses: the PRISMA statement. BMJ.

[25] Richard Landis J, Koch GG (1977). The measurement of observer agreement for categorical data. Biometrics.

[26] CASP Checklists.

[27] Long HA, French DP, Brooks JM (2020). Optimising the value of the critical appraisal skills programme (CASP) tool for quality appraisal in qualitative evidence synthesis. Res Method Med Health Sci.

[28] Boeije HR, van Wesel F, Alisic E (2011). Making a difference: towards a method for weighing the evidence in a qualitative synthesis. J Eval Clin Pract.

[29] Chrastina J (2018). Meta-synthesis of qualitative studies: background, methodology and applications. Proceedings of the NORDSCI International Conference.

[30] Braun V, Clarke V (2013). Successful Qualitative Research: A Practical Guide for Beginners.

[31] Thomas J, Harden A (2008). Methods for the thematic synthesis of qualitative research in systematic reviews. BMC Med Res Methodol.

[32] Greer B, Newbery K, Cella M, Wykes T (2019). Predicting inpatient aggression in forensic services using remote monitoring technology: qualitative study of staff perspectives. J Med Internet Res.

[33] Ng A, Kornfield R, Schueller SM, Zalta AK, Brennan M, Reddy M (2019). Provider perspectives on integrating sensor-captured patient-generated data in mental health care. Proc ACM Hum Comput Interact.

[34] Blease C, Locher C, Leon-Carlyle M, Doraiswamy M (2020). Artificial intelligence and the future of psychiatry: qualitative findings from a global physician survey. Digit Health.

[35] Thenral M, Annamalai A (2021). Challenges of building, deploying, and using AI-enabled telepsychiatry platforms for clinical practice among urban Indians: a qualitative study. Indian J Psychol Med.

[36] Dawoodbhoy FM, Delaney J, Cecula P, Yu J, Peacock I, Tan J, Cox B (2021). AI in patient flow: applications of artificial intelligence to improve patient flow in NHS acute mental health inpatient units. Heliyon.

[37] Götzl C, Hiller S, Rauschenberg C, Schick A, Fechtelpeter J, Fischer Abaigar U, Koppe G, Durstewitz D, Reininghaus U, Krumm S (2022). Artificial intelligence-informed mobile mental health apps for young people: a mixed-methods approach on users' and stakeholders' perspectives. Child Adolesc Psychiatry Ment Health.

[38] Reis L, Maier C (2022). Artificial intelligence in mental health: a qualitative expert study on realistic application scenarios and future directions.

[39] Barnett I, Torous J, Staples P, Sandoval L, Keshavan M, Onnela JP (2018). Relapse prediction in schizophrenia through digital phenotyping: a pilot study. Neuropsychopharmacology.

[40] Goddard K, Roudsari A, Wyatt JC (2012). Automation bias: a systematic review of frequency, effect mediators, and mitigators. J Am Med Inform Assoc.

[41] Fry H (2018). Hello World: How to be Human in the Age of the Machine.

[42] Matthews M, Abdullah S, Gay G, Choudhury T (2014). Tracking mental well-being: balancing rich sensing and patient needs. Computer.

[43] Yli-Kauhaluoma S, Pantzar M (2018). Seeking connectivity to everyday health and wellness experiences: specificities and consequences of connective gaps in self-tracking data. Digit Health.

[44] Brown JE, Halpern J (2021). AI chatbots cannot replace human interactions in the pursuit of more inclusive mental healthcare. SSM Ment Health.

[45] Tong F, Lederman R, D'Alfonso S, Berry K, Bucci S (2022). Digital therapeutic alliance with fully automated mental health smartphone apps: a narrative review. Front Psychiatry.

[46] Etkin J (2016). The hidden cost of personal quantification. J Consum Res.

[47] Simpson CC, Mazzeo SE (2017). Calorie counting and fitness tracking technology: associations with eating disorder symptomatology. Eat Behav.

[48] Ryan J, Edney S, Maher C (2019). Anxious or empowered? a cross-sectional study exploring how wearable activity trackers make their owners feel. BMC Psychol.

[49] McKinney P, Cox AM, Sbaffi L (2019). Information literacy in food and activity tracking among parkrunners, people with type 2 diabetes, and people with irritable bowel syndrome: exploratory study. J Med Internet Res.

[50] Lovejoy CA, Buch V, Maruthappu M (2019). Technology and mental health: the role of artificial intelligence. Eur Psychiatry.

[51] Lim HM, Teo CH, Ng CJ, Chiew TK, Ng WL, Abdullah A, Abdul Hadi H, Liew CS, Chan CS (2021). An automated patient self-monitoring system to reduce health care system burden during the COVID-19 pandemic in Malaysia: development and implementation study. JMIR Med Inform.

[52] Kelly JT, Campbell KL, Gong E, Scuffham P (2020). The internet of things: impact and implications for health care delivery. J Med Internet Res.

[53] Jalali MS, Kaiser JP, Siegel M, Madnick S (2019). The internet of things promises new benefits and risks: a systematic analysis of adoption dynamics of IoT products. IEEE Secur Priv.

[54] Lee EE, Torous J, De Choudhury M, Depp CA, Graham SA, Kim HC, Paulus MP, Krystal JH, Jeste DV (2021). Artificial intelligence for mental health care: clinical applications, barriers, facilitators, and artificial wisdom. Biol Psychiatry Cogn Neurosci Neuroimaging.

[55] Malterud K (2019). Qualitative Metasynthesis: A Research Method for Medicine and Health Sciences.

[56] Sim J, Mengshoel AM (2022). Metasynthesis: issues of empirical and theoretical context. Qual Quant.

[57] Vaismoradi M, Turunen H, Bondas T (2013). Content analysis and thematic analysis: implications for conducting a qualitative descriptive study. Nurs Health Sci.

[58] Braun V, Clarke V (2006). Using thematic analysis in psychology. Qual Res Psychol.

[59] Finfgeld-Connett D (2010). Generalizability and transferability of meta-synthesis research findings. J Adv Nurs.

[60] Gama F, Tyskbo D, Nygren J, Barlow J, Reed J, Svedberg P (2022). Implementation frameworks for artificial intelligence translation into health care practice: scoping review. J Med Internet Res.

